# Crystal structure of 2-oxo-2-phenyl­ethyl diiso­propyl­carbamate

**DOI:** 10.1107/S2056989021006927

**Published:** 2021-07-13

**Authors:** Viktor Martens, Helmar Görls, Wolfgang Imhof

**Affiliations:** a Institute of Integrated Natural Sciences, University Koblenz - Landau, Universitätsstr. 1, 56070 Koblenz, Germany; b Institute of Inorganic and Analytical Chemistry, Friedrich-Schiller-University Jena, Humboldtstr. 8, 07743 Jena, Germany

**Keywords:** crystal structure, urethanes, carbamates, C—H⋯O hydrogen bonds

## Abstract

In the mol­ecular structure of the title compound, C_15_H_21_NO_3_, the urethane function and the benzoyl group are almost perpendicular to each other [dihedral angle 88.97 (5)°]. In the crystal structure, infinite supra­molecular layers in the *bc* plane are formed by weak C—H⋯O hydrogen bonds.

## Abstract 2

In the mol­ecular structure of the title compound, the urethane function and the benzoyl group are almost perpendicular to each other [dihedral angle 88.97 (5)°]. In the crystal structure, infinite supra­molecular layers in the *bc* plane are formed by weak C—H⋯O hydrogen bonds.

## Chemical context   

Phenacyl and desyl compounds have been a subject of inter­est for many years due to their use as photoremovable protecting groups (PPGs) (Givens *et al.*, 2012 ▸; Kammari *et al.*, 2007 ▸; Klán *et al.*, 2013 ▸; Sheehan & Umezawa, 1973 ▸). Carbamates are used for the protection of carb­oxy­lic acids and may also act as suitable protecting groups for amines (Speckmeier *et al.*, 2018 ▸). Speckmeier and co-workers synthesized several phenacyl urethanes, but the protection of diiso­propyl­amine by a phenacyl group has not been reported so far. The title compound was synthesized according to reported routes (Speckmeier *et al.*, 2018 ▸). 
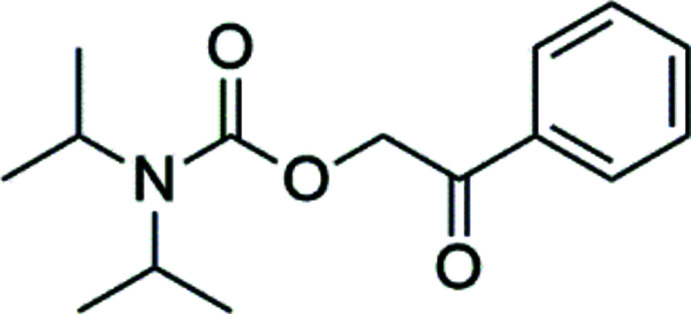


## Structural commentary   

**Figure 1:**
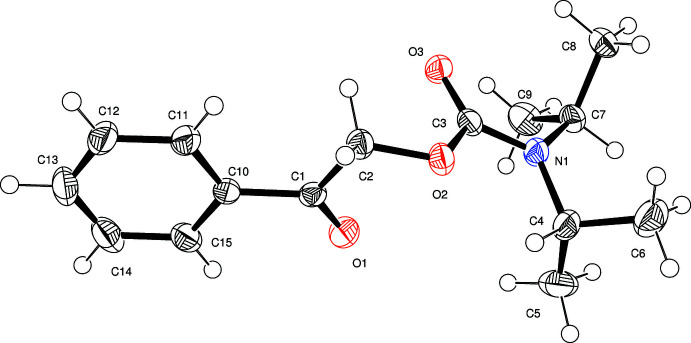
Mol­ecular structure of the title compound with displacement ellipsoids drawn at the 50% probability level.

As expected, the carbamate functional moiety (N1/C3/O3/O2) is essentially planar (maximum deviation of 0.01 Å for C3). The same is true for the benzoyl group (C1/O1/C10–C15, maximum deviation of 0.05 Å for O1). These two planes subtend a dihedral angle of 88.97 (5)° and therefore an almost perpendicular arrangement (Fig. 1 ▸). Otherwise, the bond lengths and angles are of expected values with C3—N1 [1.348 (2) Å] and C3—O2 [1.368 (2) Å] being slightly shorter than a typical C—O or C—N single bond due to the partial double-bond character of the respective bonds in a carbamate.

## Supra­molecular features   

**Figure 2:**
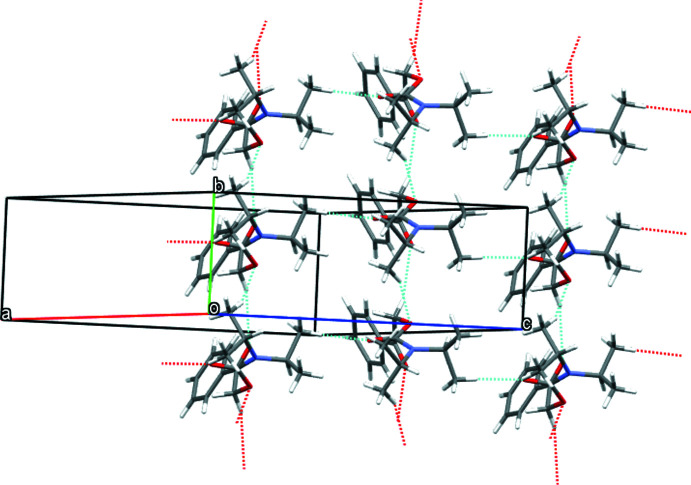
Crystal structure of the title compound showing layers of mol­ecules along the *bc* plane that are built up by C—H⋯O hydrogen bonds.

The crystal structure of the title compound features weak hydrogen bonds (Desiraju & Steiner, 2001 ▸) of the C—H⋯O type, as shown in Table 1 ▸. The inter­action C5—H5*B*⋯O3 links mol­ecules of the title compound into infinite chains parallel to the *c*-axis direction. Additional C2—H2*B*⋯O1 and C9—H9*B*⋯O2 inter­actions link these infinite chains to a supra­molecular sheet parallel to the *bc* plane (Fig. 2 ▸). The latter inter­action is accompanied by a short C9—H9*B*⋯C3 contact, which makes the contact look like a non-classical hydrogen bond towards the π-system of a C=O double bond, again showing the partial double-bond character of the respective bond.

## Database survey   

In the CSD (ConQuest Version 2020.3.0; Groom *et al.*, 2016 ▸), only one other carbamate with a CH_2_–C(O)-Ph group attached to the carbamate oxygen atom is reported (NIWQUI; Jiang *et al.*, 2019 ▸). The respective compound shows a di­ethyl­amino group and a *p*-chloro­phenyl substituent instead of the diiso­propyl­amino group and the non-substituted phenyl group in the title compound. In contrast to the title compound, the carbamate plane and the benzoyl plane are almost coplanar. The carbonyl oxygen atoms show numerous short contacts towards different C—H groups of neighboring mol­ecules, leading to a dense three-dimensional network.

## Synthesis and crystallization   

Diiso­propyl­amine (0.05 mol, 5.05 g) and 1 equiv. of cesium carbonate (0.05 mol, 16.55 g) were placed in a Schlenk tube and dissolved in anhydrous DMSO (150 mL). The tube was sealed with a septum and two balloons filled with CO_2_ were bubbled through the reaction mixture within one h while stirring. After the addition of CO_2_, 1.1 equiv. of 2-bromo-1-phenyl­ethan-1-one (0.055 mol, 10.95 g) dissolved in a small amount of DMSO was added in one portion. The consumption of 2-bromo-1-phenyl­ethan-1-one was monitored by TLC and after 30 min the reaction mixture was poured on ice to quench the reaction. After extraction with di­chloro­methane (3×), the combined organic phases were washed with brine, separated and dried over Na_2_SO_4_. The solvent was removed *in vacuo* and the crude product was recrystallized from *n*-hexa­ne/ethanol (4:1) to afford the title compound (12.90 g; 98%) as a colorless solid, m.p. 347.5°C. ^1^H NMR (500 MHz, CDCl_3_) [ppm]: δ = 7.90 (*dd*, 2H), 7.55 (*ddt*, 1H), 7.45 (*dd*, J = 8.4, 7.1 Hz, 2H), 5.33 (*s*, 2H), 3.97 (hept, 2H), 1.25 (*d*, 12H); ^13^C NMR (126 MHz, CDCl_3_) [ppm]: δ = 193.91 (*C*=O), 154.80 (N*C*=O), 134.69, 133.65, 128.84, 127.83 (*C*_ar_), 66.36 (O=*C—*O), 46.32 [(H_3_C)_2_ *C*H–], 20.99 [(H_3_ *C*)_2_CH–].

## Refinement   

Crystal data, data collection and structure refinement details are summarized in Table 2 ▸. All hydrogen atoms were placed in idealized positions (C—H = 0.95–0.99Å) and refined using a riding model with isotropic displacement parameters calculated as *U*_iso_(H) = 1.2×*U*_eq_(C) for methyl­ene and hydrogen atoms of the phenyl group or 1.5×*U*_eq_(C) for methyl groups.

## Supplementary Material

Crystal structure: contains datablock(s) I. DOI: 10.1107/S2056989021006927/zl5014sup1.cif

Structure factors: contains datablock(s) I. DOI: 10.1107/S2056989021006927/zl5014Isup2.hkl

Supporting information file. DOI: 10.1107/S2056989021006927/zl5014Isup3.cml

CCDC reference: 2094771

Additional supporting information:  crystallographic information; 3D view; checkCIF report

## Figures and Tables

**Table 1:** Hydrogen-bond geometry (Å, °)

*D*—H⋯*A*	*D*—H	H⋯*A*	*D*⋯*A*	*D*—H⋯*A*
C2—H2*B*⋯O1^i^	0.99	2.70	3.605 (2)	152
C5—H5*B*⋯O3^ii^	0.98	2.62	3.578 (2)	167
C9—H9*B*⋯O2^iii^	0.98	2.68	3.599 (2)	157

Symmetry codes: (i) x, y-1, z; (ii) x, -y+{\script{3\over 2}}, z+{\script{1\over 2}}; (iii) x, y+1, z.

**Table 2:** Experimental details

Crystal data	
Chemical formula	C_15_H_21_NO_3_
*M*_r_	263.33
Crystal system, space group	Monoclinic, *P*2_1_/*c*
Temperature (K)	133
*a*, *b*, *c* (Å)	18.4574 (8), 5.7020 (2), 14.8058 (6)
β (°)	113.468 (1)
*V* (Å^3^)	1429.33 (10)
*Z*	4
Radiation type	Mo *K*α
μ (mm^−1^)	0.09
Crystal size (mm)	0.10 × 0.10 × 0.08

Data collection	
Diffractometer	Nonius KappaCCD
Absorption correction	Multi-scan (*SADABS*; Krause *et al.*, 2015 ▸)
*T*_min_, *T*_max_	0.674, 0.746
No. of measured, independent and observed [*I* > 2σ(*I*)] reflections	13968, 3280, 2464
*R*_int_	0.040
(sin θ/λ)_max_ (Å^−1^)	0.649

Refinement	
*R*[*F* ^2^ > 2σ(*F* ^2^)], *wR*(*F* ^2^), *S*	0.049, 0.113, 1.04
No. of reflections	3280
No. of parameters	177
H-atom treatment	H-atom parameters constrained
Δρ_max_, Δρ_min_ (e Å^−3^)	0.27, −0.21

Computer programs: *COLLECT* (Nonius 1998 ▸), *DENZO* (Otwinowski & Minor, 1997 ▸), *SHELXS97* (Sheldrick, 2008 ▸), *SHELXL2018/3* (Sheldrick, 2015 ▸), *ORTEP-3* (Farrugia, 2012 ▸) and *Mercury* (Macrae *et al.*, 2020 ▸).

## supplementary crystallographic information

### Crystal data

**Table:**

C_15_H_21_NO_3_	*F*(000) = 568
*M**_r_* = 263.33	*D*_x_ = 1.224 Mg m^−^^3^
Monoclinic, *P*2_1_/*c*	Mo *K*α radiation, λ = 0.71073 Å
*a* = 18.4574 (8) Å	Cell parameters from 13968 reflections
*b* = 5.7020 (2) Å	θ = 2.8–27.5°
*c* = 14.8058 (6) Å	µ = 0.09 mm^−^^1^
β = 113.468 (1)°	*T* = 133 K
*V* = 1429.33 (10) Å^3^	Prism, colourless
*Z* = 4	0.10 × 0.10 × 0.08 mm

### Data collection

**Table:**

Nonius KappaCCD diffractometer	2464 reflections with *I* > 2σ(*I*)
phi + ω – scans	*R*_int_ = 0.040
Absorption correction: multi-scan (SADABS; Krause *et al.*, 2015)	θ_max_ = 27.5°, θ_min_ = 2.8°
*T*_min_ = 0.674, *T*_max_ = 0.746	*h* = −23→23
13968 measured reflections	*k* = −5→7
3280 independent reflections	*l* = −19→18

### Refinement

**Table:**

Refinement on *F*^2^	Secondary atom site location: difference Fourier map
Least-squares matrix: full	Hydrogen site location: inferred from neighbouring sites
*R*[*F*^2^ > 2σ(*F*^2^)] = 0.049	H-atom parameters constrained
*wR*(*F*^2^) = 0.113	*w* = 1/[σ^2^(*F*_o_^2^) + (0.0368*P*)^2^ + 0.6743*P*] where *P* = (*F*_o_^2^ + 2*F*_c_^2^)/3
*S* = 1.04	(Δ/σ)_max_ < 0.001
3280 reflections	Δρ_max_ = 0.27 e Å^−^^3^
177 parameters	Δρ_min_ = −0.21 e Å^−^^3^
0 restraints	Extinction correction: SHELXL2018/3 (Sheldrick 2015)
Primary atom site location: structure-invariant direct methods	Extinction coefficient: 0.0093 (16)

### Special details

**Table:**

Geometry. All esds (except the esd in the dihedral angle between two l.s. planes) are estimated using the full covariance matrix. The cell esds are taken into account individually in the estimation of esds in distances, angles and torsion angles; correlations between esds in cell parameters are only used when they are defined by crystal symmetry. An approximate (isotropic) treatment of cell esds is used for estimating esds involving l.s. planes.

### Fractional atomic coordinates and isotropic or equivalent isotropic displacement parameters (Å^2^)

**Table:**

	*x*	*y*	*z*	*U*_iso_*/*U*_eq_	
O1	0.38605 (6)	0.7949 (2)	0.38826 (9)	0.0338 (3)	
O2	0.27936 (6)	0.4581 (2)	0.32610 (8)	0.0280 (3)	
O3	0.24228 (6)	0.6202 (2)	0.17458 (8)	0.0285 (3)	
N1	0.17054 (7)	0.6836 (3)	0.26709 (9)	0.0285 (3)	
C1	0.41005 (8)	0.6137 (3)	0.36687 (11)	0.0234 (3)	
C2	0.35448 (8)	0.4079 (3)	0.32488 (12)	0.0257 (3)	
H2A	0.348514	0.377390	0.256450	0.031*	
H2B	0.376866	0.265560	0.364461	0.031*	
C3	0.23136 (8)	0.5945 (3)	0.24965 (11)	0.0249 (3)	
C4	0.16762 (9)	0.6747 (4)	0.36534 (12)	0.0371 (4)	
H4	0.211633	0.570586	0.407718	0.044*	
C5	0.18152 (13)	0.9155 (5)	0.41331 (15)	0.0605 (7)	
H5A	0.228794	0.985787	0.409862	0.091*	
H5B	0.188987	0.899895	0.482386	0.091*	
H5C	0.135734	1.015930	0.378631	0.091*	
C6	0.09072 (11)	0.5665 (4)	0.36026 (15)	0.0452 (5)	
H6A	0.046572	0.670305	0.322993	0.068*	
H6B	0.093071	0.545940	0.427083	0.068*	
H6C	0.083000	0.413759	0.327431	0.068*	
C7	0.11069 (9)	0.8271 (3)	0.19039 (11)	0.0278 (4)	
H7	0.074178	0.887235	0.220031	0.033*	
C8	0.06055 (9)	0.6780 (3)	0.10240 (12)	0.0319 (4)	
H8A	0.019602	0.775918	0.054376	0.048*	
H8B	0.035643	0.551161	0.124422	0.048*	
H8C	0.094128	0.610990	0.071729	0.048*	
C9	0.14496 (11)	1.0413 (3)	0.16096 (14)	0.0405 (5)	
H9A	0.176297	0.991762	0.124230	0.061*	
H9B	0.178789	1.126741	0.220191	0.061*	
H9C	0.101940	1.143637	0.119443	0.061*	
C10	0.49370 (8)	0.5861 (3)	0.37810 (11)	0.0248 (3)	
C11	0.51983 (9)	0.3863 (3)	0.34607 (12)	0.0315 (4)	
H11	0.484354	0.260546	0.317232	0.038*	
C12	0.59781 (10)	0.3710 (4)	0.35634 (13)	0.0420 (5)	
H12	0.615586	0.234665	0.334388	0.050*	
C13	0.64925 (10)	0.5519 (4)	0.39804 (13)	0.0478 (6)	
H13	0.702529	0.540331	0.404875	0.057*	
C14	0.62389 (10)	0.7512 (4)	0.43024 (13)	0.0437 (5)	
H14	0.659736	0.876252	0.458872	0.052*	
C15	0.54637 (9)	0.7686 (3)	0.42078 (12)	0.0327 (4)	
H15	0.529143	0.904967	0.443406	0.039*	

### Atomic displacement parameters (Å^2^)

**Table:**

	*U*^11^	*U*^22^	*U*^33^	*U*^12^	*U*^13^	*U*^23^
O1	0.0316 (6)	0.0311 (6)	0.0386 (7)	0.0050 (5)	0.0140 (5)	−0.0076 (5)
O2	0.0204 (5)	0.0369 (6)	0.0260 (6)	0.0011 (5)	0.0087 (4)	0.0069 (5)
O3	0.0261 (5)	0.0375 (7)	0.0229 (6)	0.0009 (5)	0.0108 (4)	0.0031 (5)
N1	0.0201 (6)	0.0433 (8)	0.0219 (7)	0.0018 (6)	0.0081 (5)	0.0037 (6)
C1	0.0248 (7)	0.0269 (8)	0.0182 (7)	0.0041 (6)	0.0080 (6)	0.0014 (6)
C2	0.0227 (7)	0.0278 (8)	0.0264 (8)	0.0023 (6)	0.0095 (6)	0.0021 (6)
C3	0.0199 (7)	0.0310 (8)	0.0211 (7)	−0.0038 (6)	0.0055 (6)	0.0007 (6)
C4	0.0257 (8)	0.0627 (13)	0.0251 (8)	0.0034 (8)	0.0125 (7)	0.0044 (8)
C5	0.0611 (13)	0.0909 (18)	0.0325 (10)	−0.0377 (13)	0.0217 (10)	−0.0224 (11)
C6	0.0487 (11)	0.0512 (12)	0.0475 (11)	−0.0061 (9)	0.0314 (9)	0.0003 (9)
C7	0.0218 (7)	0.0317 (9)	0.0269 (8)	0.0026 (6)	0.0065 (6)	−0.0008 (7)
C8	0.0251 (7)	0.0352 (9)	0.0289 (8)	−0.0006 (7)	0.0041 (7)	−0.0019 (7)
C9	0.0418 (10)	0.0327 (10)	0.0417 (10)	−0.0035 (8)	0.0108 (8)	0.0002 (8)
C10	0.0227 (7)	0.0335 (9)	0.0174 (7)	0.0036 (6)	0.0069 (6)	0.0035 (6)
C11	0.0278 (8)	0.0404 (10)	0.0257 (8)	0.0066 (7)	0.0101 (7)	0.0001 (7)
C12	0.0323 (9)	0.0672 (13)	0.0276 (9)	0.0201 (9)	0.0133 (7)	0.0047 (9)
C13	0.0209 (8)	0.0923 (17)	0.0311 (10)	0.0091 (10)	0.0111 (7)	0.0159 (10)
C14	0.0265 (8)	0.0666 (14)	0.0318 (10)	−0.0123 (9)	0.0051 (7)	0.0068 (9)
C15	0.0288 (8)	0.0404 (10)	0.0252 (8)	−0.0038 (7)	0.0068 (7)	0.0003 (7)

### Geometric parameters (Å, º)

**Table:**

O1—C1	1.2149 (19)	C7—C9	1.517 (2)
O2—C3	1.3684 (18)	C7—C8	1.522 (2)
O2—C2	1.4230 (17)	C7—H7	1.0000
O3—C3	1.2148 (18)	C8—H8A	0.9800
N1—C3	1.348 (2)	C8—H8B	0.9800
N1—C7	1.4764 (19)	C8—H8C	0.9800
N1—C4	1.478 (2)	C9—H9A	0.9800
C1—C10	1.494 (2)	C9—H9B	0.9800
C1—C2	1.519 (2)	C9—H9C	0.9800
C2—H2A	0.9900	C10—C15	1.392 (2)
C2—H2B	0.9900	C10—C11	1.392 (2)
C4—C5	1.520 (3)	C11—C12	1.389 (2)
C4—C6	1.522 (2)	C11—H11	0.9500
C4—H4	1.0000	C12—C13	1.371 (3)
C5—H5A	0.9800	C12—H12	0.9500
C5—H5B	0.9800	C13—C14	1.384 (3)
C5—H5C	0.9800	C13—H13	0.9500
C6—H6A	0.9800	C14—C15	1.385 (2)
C6—H6B	0.9800	C14—H14	0.9500
C6—H6C	0.9800	C15—H15	0.9500
			
C3—O2—C2	114.64 (12)	N1—C7—C8	111.27 (13)
C3—N1—C7	119.12 (13)	C9—C7—C8	112.63 (14)
C3—N1—C4	122.37 (13)	N1—C7—H7	106.3
C7—N1—C4	117.83 (13)	C9—C7—H7	106.3
O1—C1—C10	121.89 (14)	C8—C7—H7	106.3
O1—C1—C2	120.45 (14)	C7—C8—H8A	109.5
C10—C1—C2	117.64 (13)	C7—C8—H8B	109.5
O2—C2—C1	110.00 (13)	H8A—C8—H8B	109.5
O2—C2—H2A	109.7	C7—C8—H8C	109.5
C1—C2—H2A	109.7	H8A—C8—H8C	109.5
O2—C2—H2B	109.7	H8B—C8—H8C	109.5
C1—C2—H2B	109.7	C7—C9—H9A	109.5
H2A—C2—H2B	108.2	C7—C9—H9B	109.5
O3—C3—N1	125.75 (14)	H9A—C9—H9B	109.5
O3—C3—O2	122.46 (14)	C7—C9—H9C	109.5
N1—C3—O2	111.72 (13)	H9A—C9—H9C	109.5
N1—C4—C5	111.30 (16)	H9B—C9—H9C	109.5
N1—C4—C6	111.37 (14)	C15—C10—C11	119.50 (15)
C5—C4—C6	111.69 (16)	C15—C10—C1	118.42 (15)
N1—C4—H4	107.4	C11—C10—C1	122.07 (14)
C5—C4—H4	107.4	C12—C11—C10	119.92 (17)
C6—C4—H4	107.4	C12—C11—H11	120.0
C4—C5—H5A	109.5	C10—C11—H11	120.0
C4—C5—H5B	109.5	C13—C12—C11	120.25 (18)
H5A—C5—H5B	109.5	C13—C12—H12	119.9
C4—C5—H5C	109.5	C11—C12—H12	119.9
H5A—C5—H5C	109.5	C12—C13—C14	120.29 (16)
H5B—C5—H5C	109.5	C12—C13—H13	119.9
C4—C6—H6A	109.5	C14—C13—H13	119.9
C4—C6—H6B	109.5	C13—C14—C15	120.11 (18)
H6A—C6—H6B	109.5	C13—C14—H14	119.9
C4—C6—H6C	109.5	C15—C14—H14	119.9
H6A—C6—H6C	109.5	C14—C15—C10	119.93 (18)
H6B—C6—H6C	109.5	C14—C15—H15	120.0
N1—C7—C9	113.40 (13)	C10—C15—H15	120.0

### Hydrogen-bond geometry (Å, º)

**Table:**

*D*—H···*A*	*D*—H	H···*A*	*D*···*A*	*D*—H···*A*
C2—H2*B*···O1^i^	0.99	2.70	3.605 (2)	152
C5—H5*B*···O3^ii^	0.98	2.62	3.578 (2)	167
C9—H9*B*···O2^iii^	0.98	2.68	3.599 (2)	157

Symmetry codes: (i) *x*, *y*−1, *z*; (ii) *x*, −*y*+3/2, *z*+1/2; (iii) *x*, *y*+1, *z*.
